# Human rights education for nursing students: A scoping review

**DOI:** 10.1177/09697330241284096

**Published:** 2024-09-25

**Authors:** Elisabeth Irene Karlsen Dogan, Laura Terragni, Anne Raustøl

**Affiliations:** 87368 VID Specialized University; 158935 OsloMet; 87368 VID Specialized University

**Keywords:** Human rights, human rights education, nursing education, nursing ethics, nursing student, scoping review

## Abstract

**Background:** Human rights are an important part of nursing care, and nurses deal with human rights matter daily. Despite their relevance and acknowledgement of their importance, human rights issues remain limited in nursing education. **Aim:** The study’s aim was to describe how human rights education has been addressed in nursing education. **Method:** A scoping review was conducted according to the Preferred Reporting Items for Scoping reviews (PRISMA-ScR) and Joanna Briggs Institute (JBI) recommendations. The search was conducted in March 2023, with an updated search in February 2024. We searched in the following databases provided by EBSCO: Academic Search Elite, CINAHL, Education Source, ERIC, ScienceDirect and MEDLINE. Additionally, we also searched in Embase via Ovia and Scopus. The papers were screened for eligibility by title, abstract and full text independently by at least two reviewers, and the whole research team was involved in this process. **Ethical considerations:** The scoping review was guided by ethical conduct and scientific guidelines. **Findings:** Nine papers matched the inclusion criteria. Three thematic groups were identified: (a) focus of human rights education, (b) the learning design of the coursework and (c) learning outcomes in human rights education. **Conclusion:** Human rights education can benefit from being tailored to the students’ experiences and including voices from the rights-holders. Bringing in the students’ experiences and rights-holders’ voices can enable self-reflection and discussion regarding human rights concerns. Hence, if the intention is to support nursing students to develop an awareness and act upon human rights concerns, the curriculum ought to include opportunities for reflection and discussion around human rights concerns and experiences in the students’ own context.

## Introduction

Human rights are an important part of nursing care, and nurses deal with human rights issues daily.^1,2^ Human rights are referred to in the International Code of Ethics of Nursing, in the position statement of the International Council of Nurses (ICN)—entitled “Nurses and Human Rights”—and in national guidelines.^2–4^ The ICN endorses the Universal Declaration of Human Rights (UDHR) and the International Bill of Human Rights,^3^ underlining the inclusion of human rights norms in nursing care. As the ICN states in its Code of Ethics, “Nurses promote an environment in which the human rights, values, customs, and spiritual beliefs of the individual, family and community are respected.”^2^ Moreover, in “Nurses and Human Rights,” the ICN calls on nursing to be interpreted within the framework of international human rights agreements; they also state in the concluding remarks that human rights issues and the nursing role should be included at all levels of nursing education programmes.^3^ This is in line with the ICN Code of Ethics, which address the importance that educators ensure human rights content be addressed in the curricula.^2^

### Human rights

Human rights can be viewed from different perspectives and traditions.^5,6^ Ife has categorized three traditions of human rights thinking that has been addressed in the literature: the natural rights tradition, the legal and stated obligations tradition, and the constructed rights tradition.^6^

The natural rights tradition assumes that we are born with human rights. Earlier natural theorists, such as philosopher John Locke, have argued that human or “natural” rights are non- visible properties of personhood,^7^ and to understand our rights we must understand our humanity.^6^ There is wide consensus that human rights rest on a life with dignity and are thus a broader concept than human survival.^8^ As Donnelly and Whelan^8^ state, “we have human rights not to what we need naturally as animals for survival but to what we need for a life of dignity” (p. 24). According to this tradition, human rights are literally the rights one has because one is human.^9^

As the natural rights tradition views human rights as “given,” the tradition of legal rights views human rights as “agreed upon.”^5^ This tradition suggests that our rights exist only to the extent that they are respected, protected, guaranteed, or realized because of state action. Moreover, if we want to know what our human rights are, we investigate not the essence of our humanity, but rather the combinations of laws, conventions and government programmes.^6^

In the tradition of constructed rights, less emphasis is put on rights as existing in some objective sense, but rather looks at how people, either individually or collectively, define human rights.^6^ In this regard, human rights are seen as transformative and fought for. This tradition also emphasizes how rights are defined by people themselves, rather than theologians and philosophers (natural rights tradition) or lawyers and politicians (legal rights tradition).^6^ Approaching human rights as constructed rights means that human rights are constructed through human interaction and dialog around what should constitute a common or shared humanity. Here, then, human rights are not static, but will vary over time in different contexts and cultures.^6^

### Human rights education

Human rights education was initially introduced in 1948, via the Universal Declaration of Human Rights (UDHR).^10^ Article 26 identifies both the right to education, and the right to education directed towards the full development “of respect for human rights and fundamental freedoms.” Since then, many antecedents have been made to human rights education from non-governmental organizations (NGOs), community-based work and through individual initiatives. In the aftermath of the UN World Conference on Human Rights in Vienna (1993),^11^ seen as a watershed within human rights education; The period from 1995 to 2004 was labeled as the decade of human rights education, in which activists, policymakers, governmental representatives and educators were all engaged in the discussion (Bajaj, 2017).^12^ Human rights education relies on translating human rights policy into local contexts.^13^ While there are a variety of approaches to human rights education, varying in content, scope and comprehension, there is broader agreement relating to its core elements.^12^ Firstly, human rights education must include both *processes* and *content.*^12,14^ Secondly, human rights education must entail goals related to the content, values and actions aimed at promoting human rights.^14^ Moreover, human rights education does not merely mean transferring knowledge related to human rights in an educational context, but involves enhancing students’ ability to promote, defend and apply human rights in daily life and practice.^15^ Further embedded in human rights education is the cultivation of attitudes and behaviors required to promote and justify human rights for all.^15^

Today, there are broad international discourses on the value of human rights education for developing sustainable practices, respecting human rights, ensuring social justice and protecting human dignity.^13,16,17^ Moreover, Backan and Fitchett^18^ have underlined its importance for a deeper understanding within the health field as to what constitutes the right to health and to recognize how it adds value in practice.^18^ However, 20 years after the emergence of the health and human rights movement,^19,20^ the concern remains that human rights education is marginal in health education, including nursing education.^21,22^

### Human rights education in the nursing field

The importance of human rights education in the nursing field has long been emphasized.^16,23^ The curricula in health professions and in nursing has already embraced the issues of ethics and justice. However, the lack of awareness and knowledge about human rights for nurses and nursing students has been highlighted. A study from Turkey identified the need for human rights to be included in continuing nursing education, as it found a lack of awareness of human rights among midwifes and nurses.^24^ In a study from India, nursing students were found to have limited knowledge of human rights for mentally ill patients.^25^ This is in line with a more recent study from Nigeria,^26^ which found limitations concerning the mental health care knowledge among nursing students; the authors recommend that nursing curricula should therefore include sufficient education regarding the human rights of people with mental illness. Another study from Turkey, this one among nursing students, emphasized the need for nursing education to increase and enrich the information provided about human rights regarding children.^27^

In a more recent review of human rights education in patient care, the concern is raised in regards to the importance of discussing the nature of human rights education and of examining its potential for impacting patient care.^28^ In line with this, Pfendt^21^ indicates that there is minimal literature in the field of nursing addressing *how* to incorporate human rights issues into nursing curricula and stresses the importance of looking into the impact human rights education have on nursing students. Consequently, a deeper understanding of how human rights education has been addressed in nursing education seems timely and of importance. Therefore, in this study we aim to explore *how* human rights education has been conducted and contextualized in nursing education.

## Method

### Design

Given our aim to investigate human rights education in the field of nursing education, we chose to conduct a scoping review, as this method is suitable to explore a breadth of research; identify the types of evidence available in a given field (as a precursor to a systematic review); identify and analyze knowledge gaps; and examine how research is conducted on a certain topic or field.^29^ We approached our scoping review using the five-stage framework proposed by Arksey and O’Malley^30^: (a) identifying the research question, (b) identifying relevant studies, (c) selecting the studies, (d) charting the data and (e) collating, summarizing and reporting results. In addition, we further incorporated the methodological guidance of Peters and colleagues regarding evidence screening and selection, data extraction, analysis and presentation of results.^29,31^ The scoping review was conducted according to the Preferred Reporting Items for Scoping Reviews (PRISMA-ScR) and JBI recommendations.^32,33^

### Identifying the research question

The research question investigated in this scoping review was “How has human rights education been conducted and contextualized in nursing education?”

### Identifying relevant studies

#### Search strategy

A systematic search was conducted in March 2023 and February 2024 by a specialist librarian. Based on our investigative aim and research question, we selected the following EBSCO databases: Academic Search Elite, CINAHL, Education source, ERIC, ScienceDirect and MEDLINE. Additionally, we also searched in Embase via Ovid and Scopus. The search strategy contained subject terms and text words describing the main concepts in this review: namely, “human rights education” and “nursing education.” The search terms within the same concept were combined with the Boolean operator OR, before combining the words from each concept with AND. All the authors and a specialist librarian cooperated to build the search terms. An example of the search strategy is included in Table 1.

**Table 1.** Search strategy.

PCC	
Population OR context	Nursing education OR nursing students OR undergraduate nursing students OR nursing training program OR nursing schools OR nursing practice
Concept	AND
	Human rights OR human rights education OR human rights-based approach OR human right rights perspective OR right to health

#### Data inclusion and exclusion criteria

According to our inclusion and exclusion criteria, the inclusion of records was limited to qualitative, quantitative and mixed-methods studies, as well as descriptive educational interventions regarding the phenomenon; all had to be published in peer-reviewed journals. The phenomenon of interest was studies of human rights education in nursing education. The chosen language was English. The exclusion criteria consisted of publications in languages other than English and non-original research publications (editorials, reports, comments, letters, abstracts from conferences, books, literature reviews, theses, concept analyses, papers that had not been published in peer-reviewed journals). Gray literature was excluded, as we wanted to conduct a review as a precursor to a systematic review, and also look into how research is conducted on a certain topic or field. According to our research question, we wanted to gain knowledge and insight of how human rights education has been conducted and contextualized in nursing education within research. As far as we know, there are few if any studies that have investigated this (See Table 2).

**Table 2.** Inclusion and exclusion criteria.

	Inclusion criteria	Exclusion criteria
Type of study	Qualitative, quantitative and mixed-methods studies, as well as descriptive educational interventions on the phenomenon; all had to be published in peer-reviewed journals	Editorials, reports, comments, letters, abstracts from conferences, books, literature reviews, theses, concept analyses, papers that are not published in peer-reviewed journals
Time period	All	Non-specific date or year
Language	English	Any other language
Type of education programme in which the study or intervention is carried out	Bachelor’s degree/undergraduate nursing programmes	Other professional education programmes, interdisciplinary programmes
	Postgraduate nursing programmes	
	Studies that report on nursing and other programmes	
Phenomenon of interest	Studies of human rights education in nursing education	Studies that are not related to education or human rights

### Selecting the studies

Duplicates were removed using the EndNote software programme. Records were uploaded to the Rayyan web application with “blind-on” to manage the review process. The records were divided into two files. At the beginning of the screening process, the whole research team independently pilot tested the selection process and screened the same 100 records.^29^ This was done to facilitate further discussion about which type of papers to include and the inclusion and exclusion criteria, given the broad research question (typical of scoping reviews). This helped to enhance consistency in the selection process.^29^ The next step in the screening involved independently screening the titles and abstracts of half the records by the whole research team; the team then met again to reach consensus regarding the included records. The rest of the records were screened independently by the first and last authors. The inclusion of the papers was an iterative process, and all conflicts were resolved by consensus in the group, to reduce bias. Records were excluded from the review if they did not meet the inclusion criteria.

### Charting the data

The data were charted in a table including key information relevant to our research question. This was done in accordance with the JBI recommendations for scoping reviews, which state that the purpose is to identify, characterize, code and summarize research evidence in relation to the specific topic.^33^ The data chart includes the studies’ aim, methodology, participants, focus of the human rights education, learning design and key findings relevant to human rights education in nursing (See Table 3).

**Table 3.** Authors, year, country, aim, methodology, participants, focus of the human rights education, learning design and findings.

Authors, year, country	Aim	Methodology and participants	Focus of the human rights education	Learning design	Findings
Allan and Luders (2021) Scotland, United Kingdom	To raise awareness of the importance of children’s human rights	Descriptive and reflective design	Children’s rights	A model was developed through direct partnership with representatives of the Children’s Parliament in Scotland. This model facilitated the inclusion of children and young people’s opinions in the co-design of postgraduate nurse education, in order to incorporate children and young people’s human rights and opinions and improve the delivery of the school nurse service	Children and young people have clear opinions and views when consulted in an inclusive, age-appropriate way through human rights-based participation
		40 children			
Chamberlain (2001) United Kingdom	The teaching of human rights in nursing courses in the United Kingdom	Quantitative	Human rights education in the United Kingdom in general	Human rights were mainly taught as part of courses in ethics and law. The topics that were taught by all the teachers were values underlying human rights; ethical rights of nurses and patients/clients; and rights of particular groups. Most of the teachers also included as topics (a) the role of the nurse as advocate in safeguarding human rights and (b) Article 12 in the ICESR (the right to health)	The students had difficulties in understanding the relevance that human rights had for practice, although they were introduced to topics in human rights
		Survey (*n* = 51)			
		Teachers in nursing education			
Dogan, Terragni et al. (2022) Norway	Investigates the development of coursework on nutritional care with a human rights perspective in a nursing programme and draws upon reflections and lessons learned	Qualitative educational design research	Older adults’ right to food older	The coursework, developed in two rounds through educational design research, combined on-campus learning and clinical placement in nursing homes. The coursework included ethical challenges related to food, nutrition, diet and meal situations; structural challenges related to nutritional care in practice; roles in nutritional care; and handling challenges around the right to food in nursing homes. Reflection and discussion was facilitated around ethical challenges and rights violations with their supervisor and fellow students during their placement	A human rights perspective enabled students to give meaning to nutritional care beyond understanding food as a basic physical need. Incorporating human rights in nursing education can support nursing students and nurses in recognizing and addressing ethical and structural challenges and being able to fulfill the right to food for patients
		18 nursing students in multistage focus groups	Nutrition		
		25 students in focus group, written assignments	Learning through experiences		
Garner (2022) United States	Nursing students enrolled in an undergraduate bachelor of Science in nursing research course went to a holocaust and human rights museum to help them become “upstanders” for health equity	Descriptive and reflective design	Structural racism in health	Nursing students enrolled in an undergraduate bachelor of Science in nursing research course were taken on a field trip to a holocaust and human rights museum as a nontraditional experiential approach to build “upstanders” for health equity. Debriefing session were then held in class, and the students used their experiences to identify a topic and relevant research papers in which nurses advocate for health equity for vulnerable populations	Students used their experiences to inspire research to advocate for health equity for vulnerable populations and disseminated their research at local, national and international conferences
		625 nursing students	Health inequities		Embedding an immersive experience and related assignments is one strategy towards dismantling structural racism in health care
London and Baldwin-Ragaven (2008) South Africa, United States	This article draws on the authors’ experiences organizing and leading courses in human rights for health professional educators	Descriptive and reflective design	Human rights derived from South Africa’s Constitution	The authors stress that experiential learning seems most effective to deal with human rights concerns. Teaching and pedagogical approaches that promote self-reflection and critical thinking, such as case studies, site visits, roleplay and small groups discussion, are therefore of importance in human rights education	The authors emphasize that (a) nurses must be able to actively empower to achieve their rights; (b) as ethical obligations can bring nurses into conflict if they protect human rights, this tension should be reflected in the teaching; and (c) as the relationship between human rights and ethical codes are not always clear, dual loyalty must therefore be addressed in education
Mayers (2007) South Africa	To describe the introduction of a module in health and human rights into a postgraduate diploma curriculum for registered nurses	Descriptive and reflective design	Human rights concerns among health professionals	The module is divided into sessions of two to 3 hours. The themes included in the module attempt to provide the student with an overview of the issues while remaining practical by using case studies and practical assignments. In an introductory session, students are introduced to the core principles of human rights and given a brief background on the development of human rights in South Africa. The major codes and documentation relating to human rights are examined	Curricula which include human rights as part of the course content should ensure that the learning context is taken into consideration, and that learners’ personal backgrounds and experiences are respected. Learners enter such a course with a wealth of personal understanding and experience, and the nurse educator should be skilled in order to assist the students to engage with their own experiences as they engage with the new content
		Postgraduate nursing students	Individual and community health		
(Okenwa-Emegwa and Eriksson (2020) Sweden	To present lessons learned from incorporating roleplay about forced migration in inclusive nursing classrooms	Qualitative	Forced migration	One of the teaching-learning activities is an educational roleplay led by three to four volunteers from the youth wing of the Swedish Red Cross Society. The roleplay is a two-hour activity comprising an introduction, the roleplay, reflection and a summary. The aim is to provide background information—like vital statistics on violence due to wars and conflicts, the people affected, the numbers of displaced persons and people on the move—by gender, age and countries of origin, among others	Findings suggest that working collaboratively in an inclusive environment may improve nursing students’ understanding of the vulnerabilities created by forced migration and to be better prepared for promoting social justice for this group in health care settings
		Data materials, notes obtained from evaluation			
		656 nursing students participated in the course			
Reyes, Padilla Zuniga et al. (2013) Nicaragua, El Salvador	Describes the efforts of one Central American non-governmental organization to include human rights-related content into reproductive health care provider training programmes in Nicaragua and El Salvador	Mixed-methods design	Reproductive health	A workshop and training course for nursing students and medical school graduates on different reproductive health and human rights topics was conducted. The workshop comprised four sessions, each covering one of the following topics: (a) The relationship between health, human rights and medical ethics; (b) the relationship between human rights and reproductive health; (c) specific ways that providers can safeguard human rights in the service delivery context; and (d) the legal processes involved in reporting a human rights violation	Health care providers are not being adequately prepared to fulfill their duty to protect and promote human rights in patient care. Exposure to educational materials and methodologies that emphasize the relationship between human rights and reproductive health may lead to changes in health care provider attitudes and behaviors that help promote and safeguard human rights in patient care
		Pre-/post-test quasi-experimental study design			
		Nursing students (*n* = 25)			
		Control group (*n* = 30)			
		Semi-structured interviews doctors (*n* = 18)			
Turan (2022) Turkey	To evaluate the effects of a “women’s–children’s rights” online educational programme (WCR-OEP) in a nursing curricula	A pre-/post-test follow-up with a control group quasi-experimental trial	Children and women’s human rights	The module included national–international declarations on women’s–children’s human rights, ways to protect women from violence and gender inequality and ways to protect children from neglect and abuse. The online course was conducted once a week for 30 min for 14 weeks. All students had the chance to ask each other and the trainer questions verbally or in writing about educational content on the chat screen	The WCR-OEP was shown to develop students’ positive attitudes towards gender roles, develop positive attitudes towards violence against women and develop positive attitudes towards reporting child abuse by health care personnel
		Fourth-year students (*n* = 62)			

### Collating, summarizing and reporting the results

We summarized the papers with regards to their country of origin, year of publication, focus of the human rights education, methodology and participants. The papers and the extracted data were read several times to identify patterns of similarities and differences regarding the research question. Through an iterative process, the authors agreed upon the thematic grouping of the papers.

### Ethical considerations

The scoping review was guided by ethical conduct and scientific guidelines.

## Results

**Figure 1.**
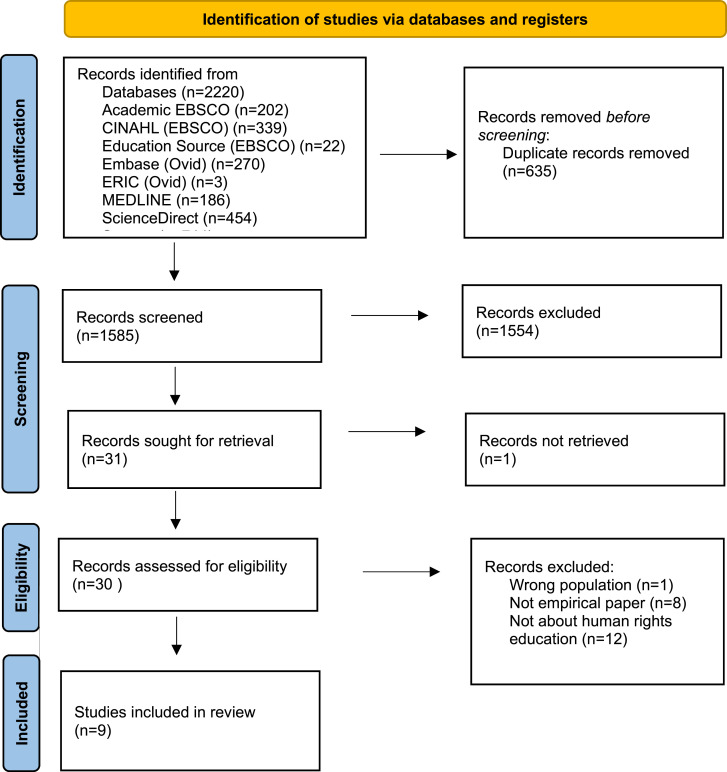
The PRISMA flowchart for this review process.

A total of 2220 records were found, and 635 duplicates were removed. Out of a total of 1585 records, 31 were selected for retrieval, and 30 were reviewed in full text by the whole research team. Figure 1 outlines the PRISMA flowchart for this review process.^33^

### Study characteristics

In total, nine studies about human rights education or similar concepts were identified from the records. The studies were conducted in South Africa,^34,35^ the United Kingdom,^36,37^ Norway,^38^ Sweden,^39^ Turkey,^40^ the United States^41^ and Nicaragua and El Salvador.^42^ The first study took place in the United Kingdom in 2001.^36^ Of the nine papers identified, four of the studies focused on nursing students in a bachelor’s programme.^38–41^

Moreover, two of the studies focused on postgraduate nursing education^34,37^; two focused on teachers and health professional educators in undergraduate education^35,36^; children and young people were included in another study^37^; and both nursing students and medical doctors were included in yet another.^42^

Four of the studies were descriptive, drawing on the authors’ reflections and experiences teaching human rights education.^34,35,37,41^ Two of the studies had a qualitative design,^38,39^ one had a quantitative design,^36^ while another used mixed-methods,^42^ and one was described as a quasi-experimental evaluation.^40^

For the two studies with a qualitative approach, one utilized data material from course evaluations, written notes and feedback from students over a 3-year period^39^; the other study drew on data material from multistage focus group interview with 18 nursing students, as well as focus groups interviews with 26 nursing students and their written assignment from clinical placement.^38^ The study that had a quantitative approach included 51 completed questionnaires from teachers in the United Kingdom representing different geographical locations.^36^ The study that utilized a mixed-methods design used both pre- and post-test evaluations with 25 nursing students and semi-structured interviews with 18 doctors to generate data.^42^ In the study with a quasi-experimental design, 62 nursing students participated in a pre- and post-test follow-up.^40^ Table 3 provides a detailed overview of the studies’ characteristics.

### Thematic groups

To answer the research question, the studies were organized into three thematic groups: (a) focus of the human rights education, (b) the learning design of the coursework and (c) learning outcomes in human rights education.

### Focus of the human rights education

The studies included different topics of human rights issues in nursing education. Two of the studies focused on children’s rights^37,40^; of these, Turan^34^ also focused on women rights. Two other studies focused on the development of human rights education in the context of South Africa.^34,35^ Another study looked into human rights courses included in the UK nursing curricula.^36^ The other topics concerning human rights included health inequities and structural racism^41^; the incorporation of human rights into reproductive health care^42^; the right to food for older adults in nursing homes^38^; and health inequities, equality and forced migration.^39^ Findings from the studies also stress the importance of having nursing educators address the issue of dual loyalty, systems and structural challenge.^35,38,41^

### Learning design of the coursework

The studies described various learning designs for human rights education. However, we found some interesting patterns. Several of the studies engaged with NGOs or other parts of civil society in the development of the learning design or the teaching of the courses. Allan and Lauders’^37^ study involved a close collaboration with the Children’s Parliament in Scotland, where children co-designed the coursework for postgraduate students in school nursing. In Okenwa-Emegwa and Eriksson’s^39^ study, the Swedish Red Cross led a roleplay; relatedly, the work of Reyes et al,^42^ is an example of cooperation with an NGO (Ipos Central America) whose focus is on cooperation with nursing and medical schools. Mayers^34^ discusses her experiences with human rights education, drawing on the use of using teaching material developed by Amnesty International. Finally, other descriptive articles emphasize the importance of involving civil society in human rights education in nursing education.^35,36^

The context and site of the teaching varied. Five of the studies took place on-campus.^34,35,39,40,42^ The articles by Garner^41^ and London and Baldwin-Ragraven^35^ describe the importance of learning designs that involve field trips: in Garner’s^41^ study, the students visit a Holocaust and human rights museum, while London and Baldwin-Ragraven^35^ discuss the importance of students visiting settings where human rights are at risk (e.g., police stations, prisons, and psychiatric institutions). One of the studies explored experiences with online teaching,^40^ while Dogan et al.’s study^38^ is the only one that involved learning about human rights in clinical placement.

Many of the studies emphasize the importance of having learning designs that build on students’ own engagement and experiences.^34,35,39^ While Chamberlain^36^ argues that human rights education is primarily addressed in general ethics and law education, our included studies demonstrate a variety of ways in which human rights are contextualized. One common strategy found in all but one study,^40^ is to combine a contextualized element (e.g., case studies), site visits,^35,41^ meetings with people who have experienced human rights violations,^34,35,39^ experiences in clinical placement,^38^ and roleplay,^39^ followed by reflective work with fellow students in small groups.

### Learning outcomes in human rights education

Some of the findings suggest that (a) health care providers are not being adequately prepared to fulfill their duty to protect and promote human rights in patient care^42^ and (b) participating in human rights education contributed to changes in health care providers’ attitudes and behavior that can help promote and safeguard human rights in patient care.^40,42^ Findings from some of the studies emphasize that human rights education helped to enhance students’ awareness of human rights violations for people in vulnerable situations.^34,38,40,42^ The findings also suggest that nursing students and health care providers who have knowledge and awareness about human rights are better equipped to both recognize and act in situations where human right are at risk and to promote human rights.^34,37,40,42^

Findings also demonstrate that human rights education can support nursing students and nurses to address structural challenges and dual loyalty.^35,38^ However, one of the studies also underscores the importance of addressing the frustration providers may experience when they face a lack of power to address complex structural barriers (e.g., resource limitations or health care systems’ infrastructure).^42^ The findings also emphasize that the students learned to advocate and stand up for health equity and social justice for vulnerable populations^33,35,39,41^ as well as to fulfill the human rights of patients.^38^ Moreover, findings suggest that human rights education can support nursing students to promote ethical practice in nursing care that is grounded within the human rights approach.^34,35,38^ Nevertheless, one study found that students had difficulties in understanding the relevance that human rights had for practice, despite being introduced to topics in human rights.^36^

## Discussion

This scoping review aimed to investigate how human rights education has been conducted and contextualized in nursing education. The database search identified 2220 citations, but only 30 studies were assessed for eligibility. Just nine of these were eligible for inclusion, which may indicate a research gap on the phenomenon of interest. Although the number of studies was small, the ways in which human rights education was taught was diverse, both regarding to topic and context. Our findings address the importance that human rights education be tailored through real-life examples and cases. Here, then, human rights are not seen as abstract theory, but as part of the challenges that can occur in daily practice in nursing care. This gave the students the opportunity to work on human rights issues of relevance. Human rights education has been criticized for being decontextualized and addressing violations far removed from the learners’ context.^14,43,44^ This may hinder students from seeing the relevance of human rights in a local context in daily care. Addressing human rights concerns through cases or in a daily context of care may enable students to move from an awareness of violations towards transformation and action.^14^

This emphasis on context is not new to human rights education. Several researchers have addressed the need for students to be aware of human rights in both daily life and in a local context.^14,17,45^ For instance, Tibbits^14^ argues that human rights learning and accountability develop through participation and socialization. As such, our findings suggest that human rights education benefits from being tailored to the students’ experiences and own context. Incorporating the students’ experiences may also facilitate self-reflection and discussion regarding human rights concerns—as emphasized in the findings and by others.^46,47^ Hence, if the intention is to support nursing students to develop an awareness of human rights, the curriculum ought to include opportunities for reflection and discussion around human rights concerns, as well as the students’ experiences in this regard.

Moreover, the findings suggest that that nursing students and health professionals who have knowledge about and awareness of human rights are better equipped to recognize and act in situations where human rights are at risk. This is in line with research including medical students that found that a human rights perspective can support students to advocate on behalf of the most vulnerable members of society.^48,49^ However, introducing a human rights perspective in nursing education is not only about awareness when human rights are potentially at stake. It was also found that introducing a human rights perspective can provide the language to articulate concerns about social justice and discrimination, as also suggested by other authors.^28,50^ This corresponds with the accountability approach from Tibbitts’ model.^14^ In this regard, human rights can provide a powerful language in which nurses can mobilize and act upon justice concerns.^50^

It was also highlighted in the studies’ findings that a human rights perspective gives rights-holders a voice. In line with this perspective, Ezer^51^ also underscores the importance of enabling the voices of the socially excluded to be heard and that human rights education should include these voices—a position also supported by others in more recent studies.^52,53^ This is not unfamiliar in nursing education and nursing care, and the importance of hearing and giving voice to patients has also been addressed by Benner et al.^54^ and Kitson et al.^55^ As patients are rights-holders, they ought to be given the opportunity to participate in social change and justice that concerns themselves.

Findings from the studies also underscore the importance of nursing educators to address the issue of dual loyalty: namely, the potential conflict between a nurses’ professional duties to their patients and their obligations to a third party, such as an employer or other authority.^3,46^ Although nurses may encounter human rights concerns daily, they may not be aware that human rights violations are occurring and, in some instances, may even be complicit in those violations. The importance of addressing dual loyalty in human rights education has also been highlighted by others, in the field of medical education.^47^ Another study emphasized the importance of both nurses advocating on behalf of their patients and of recognizing the dual role of advocacy.^56^

As addressed earlier, nurses can be important spokespersons for supporting people in vulnerable situations as they fight for their human rights. Our findings suggest the importance of not protecting merely the individual and their human rights, but also advancing policies and practices that create contextual and systemic conditions, to support patients to realize their own human rights. The importance of advocacy at both an individual level and in regards to confronting unsuitable policies or rules within the health care system has also been addressed by others.^57^ Corresponding to this is Erdman’s^16^ perspective on human rights education: that it should be about knowledge, change and justice—and that human rights is not about realizing rights in heroic and singular moments, but in the transformation of the fundamental institutions of society. According to our findings, human rights education can be appropriate for addressing challenges beyond the nurse–patient relationship, such as dual loyalty and structural challenges.

Our finding also highlights the importance of teaching nursing students to develop professional values, such as advocating for social justice and health equity, promoting dignity and respecting patients’ human rights. This is also emphasized by others.^14,16^ A human rights perspective provides a set of legally recognized and globally accepted norms for identifying systemic issues and enabling mobilization.^50^ In this regard, a human rights perspective in nursing care can further complement and work parallel to care ethics, involving ethical issues that move beyond the nurse–patient relationship.^50,58^

### Strengths and limitations

The review’s strengths are its use of an acknowledged methodological framework for conducting a scoping review, its comprehensive systematic database search and the process in which the authors independently assessed eligibility and extracted data. Furthermore, the data were analyzed and discussed by the whole research team, enhancing credibility and facilitating intersubjectivity.

Some limitations exist regarding the study’s method, however. As we only included studies published in English, some relevant papers published in other languages may have been missed. Our choice not to include gray literature may also have led to the exclusion of relevant literature. Moreover, critical appraisal of individual sources of evidence is not seen relevant to a scoping review and was deemed to be beyond the scope of this article; as such, the quality of the included studies was not assessed, and the risk of bias and the validity of the included studies are therefore unknown. A protocol published prior to the study could have enhanced further transparency and is thus seen as a limitation of the study. Finally, the studies included were few in numbers, and some of the studies were limited to looking into a short module. Although few studies indicate a research gap in this field, more studies contextualized over a longer period of time could have contributed to nuancing the findings.

### Implications for nursing practice

Moving forward, more publications on research into various areas of human rights education is needed: in particular, studies in practice settings where students and health personnel can contextualize their knowledge over a longer period of time. Indeed, as one of the reviewed studies pointed out, moving from awareness to action was more difficult to assess during a short module. Also relevant to investigate are studies that emphasize nurses’ human rights. We did not find papers addressing human rights education focusing on the human rights of nursing students and nurses themselves; as such, this should be a priority.

## Conclusion

Human rights education can benefit from being tailored to the students’ experiences and including voices from the rights-holders. Bringing in the students’ experiences and rights-holders’ voices can enable self-reflection and discussion regarding human rights concerns. Hence, if the intention is to support nursing students to develop an awareness of and act upon human rights concerns, the curriculum ought to include opportunities for reflection and discussion around human rights concerns and experiences in the students’ own context.
